# Supplementary material for: Natural variation in Glume Coverage 1 causes naked grains in sorghum
Source: Nat Commun. 2022 Feb 25;13:1068. doi: 10.1038/s41467-022-28680-3 (PMC8881591; doi:10.1038/s41467-022-28680-3)
Supplement: Supplementary file 1 — Supplementary Information [file 41467_2022_28680_MOESM1_ESM.pdf]

Natural variation in *Glume Coverage 1* causes naked grains in sorghum

Xie *et al.*

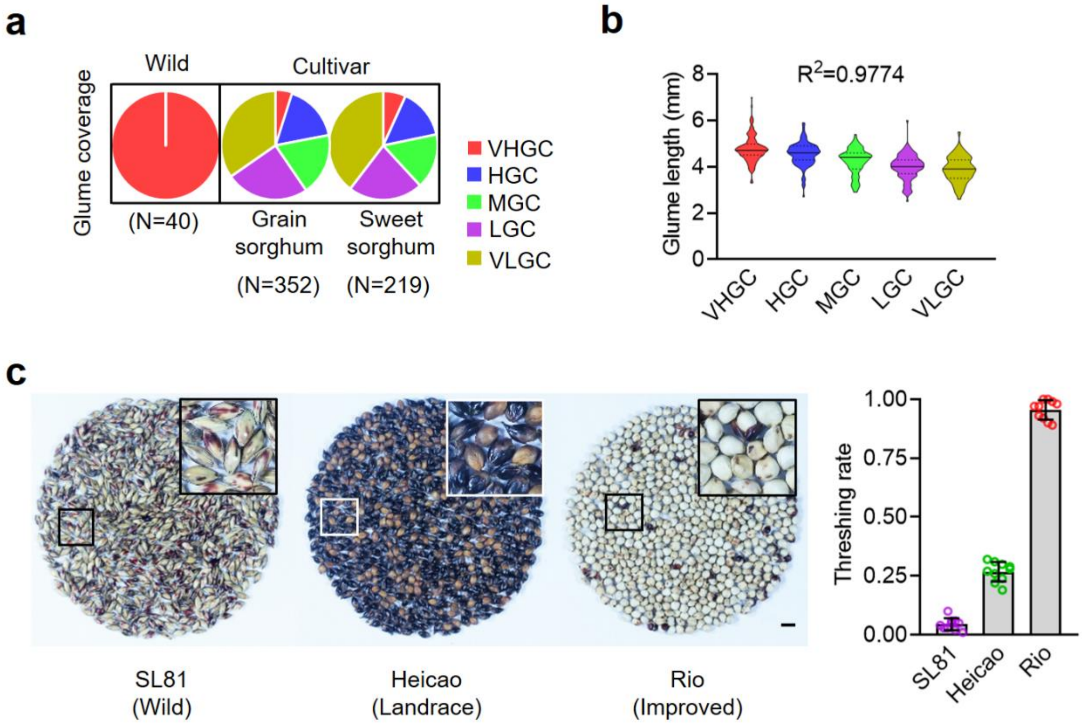

Supplementary Fig. 1. Morphology of glume coverage in diverse sorghum accessions.

a, Frequency of glume coverage in wild sorghum and sorghum cultivars (grain sorghum and sweet sorghum). VHGC, Very high glume coverage. HGC, High glume coverage. MGC, Moderate glume coverage. LGC, Low glume coverage. VLGC, Very low glume coverage. **b,** Pearson correlation analysis between glume length and glume coverage in 915 sorghum accessions. **c,** Threshing rate of the representative sorghum varieties (wild sorghum variety SL81, landrace variety Heicao and improved variety Rio) with a mechanical thresher at 1000 rpm per minute. Bar = 0.5 cm. Data are mean ± s.e.m. n = 10 biological replicates. Source data are provided as a Source Data file.

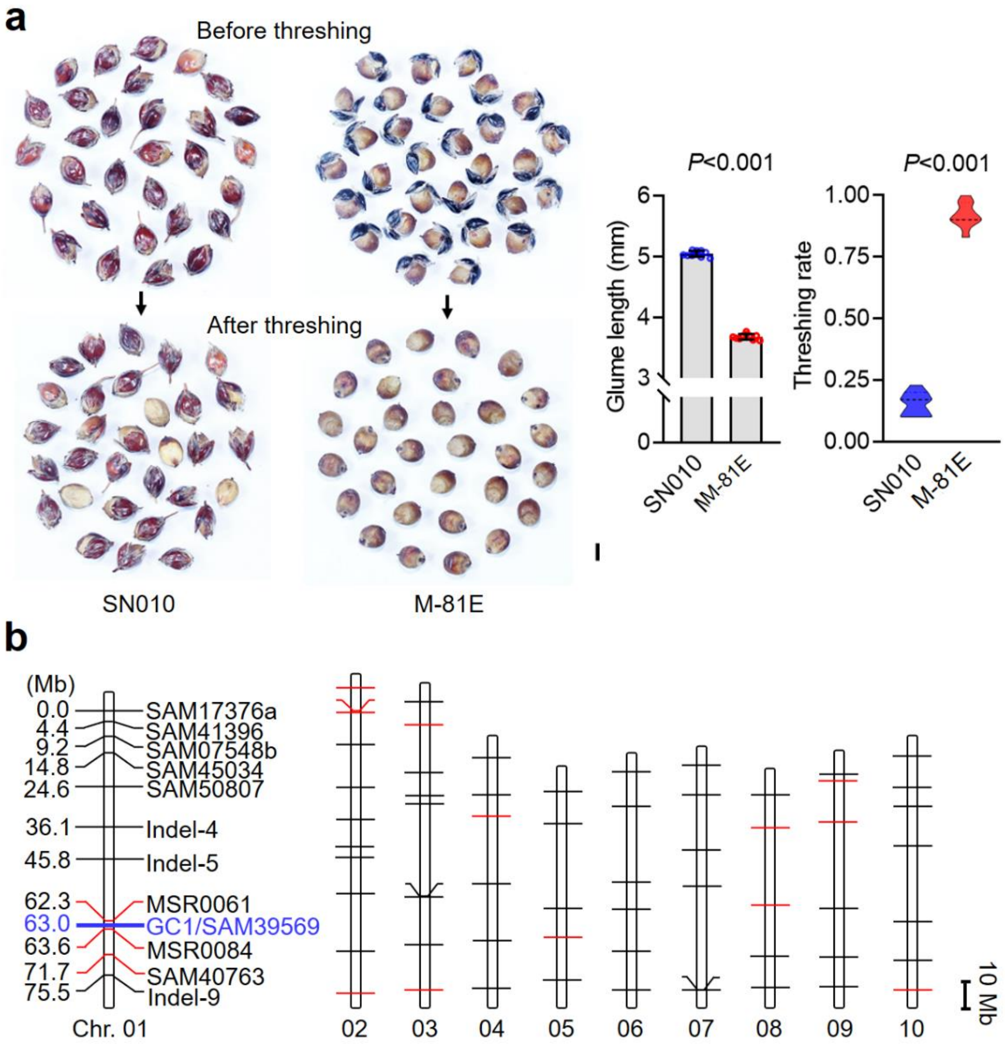

Supplementary Fig. 2. Initial map of *GC1* locus in the F_6 generation derived from the parental line SN010 and M-81E.

a, Thirty mature grains of SN010 and M-81E were threshed by a mechanical thresher with 1000 revolutions per minute. Bar = 2 mm. Statistics of glume length and threshing rate in SN010 and M-81E. Data are mean ± s.e.m. n = 10 biological replicates. *P*-values were determined by two-tailed unpaired *t*-test. **b,** Initial map of *GC1* locus in the F_6 generation. Long columns indicate the ten chromosomes. Left position show the physical distance based on the reference genome of BTx623 (version 3.1). The SSR marker *SAM39569* co-separated with *GC1* locus (highlight by blue color) was located at Chr1: 63,014 Kb (version 3.1) or Chr1: 55,886 Kb (version 1.0). Red lines show the SSR (or indel) markers have separated in the population while black lines not. Source data are provided as a Source Data file.

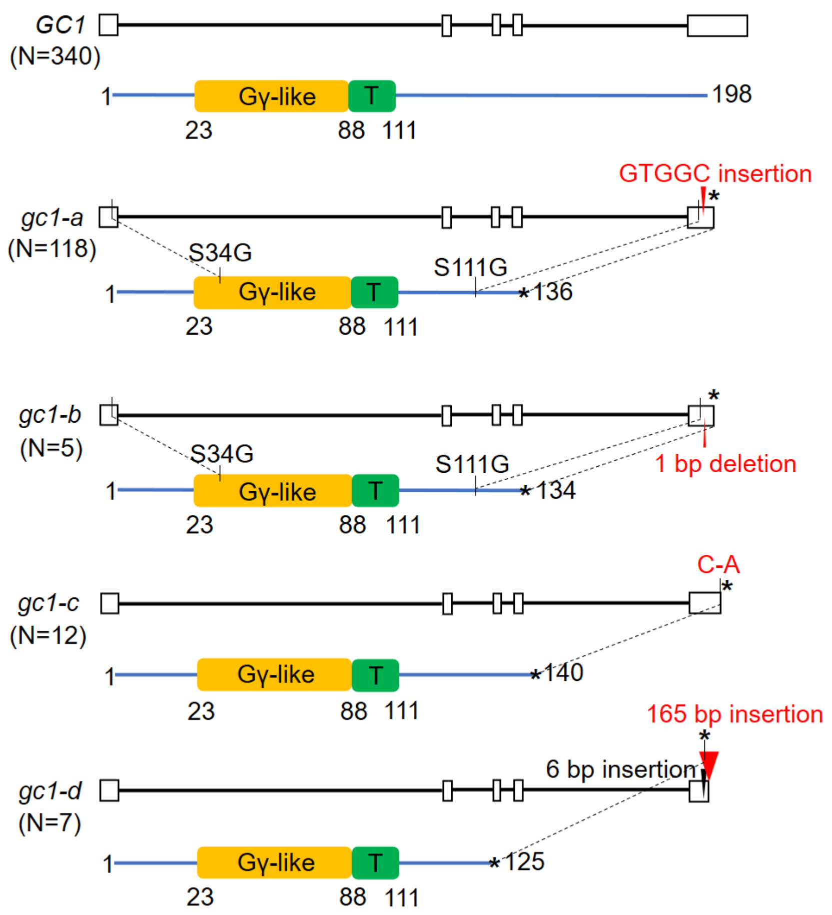

Supplementary Fig. 3. Gene and predicted peptide structural diagrams of five *GC1* haplotypes.

The top and bottom structural diagrams represent gene and predicted peptide, respectively. White box shows exon region while thick line shows intron region. The orange box shows Gγ-like subunit domain. The green box shows predicted transmembrane (T) domain. Red color indicates various mutations the fifth exon. The asterisk indicates gain of a premature stop codon. The gray line shows uncharacterized amino acids due to frameshift. N, Number of sorghum accessions.

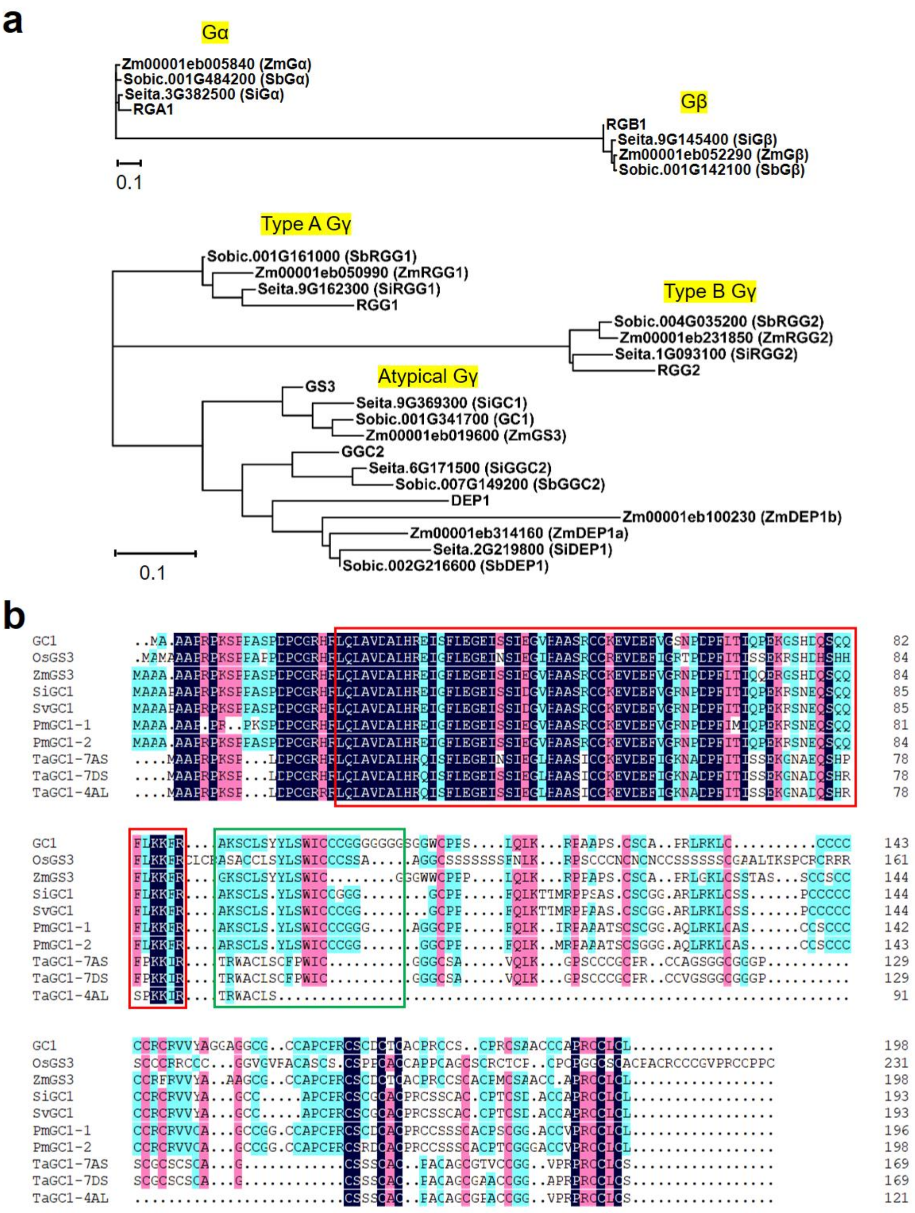

Supplementary Fig. 4. G-proteins and GC1 homologues in crops.

a, Phylogenetic tree of G-proteins in crops. The maximum-likelihood phylogenetic tree was carried out by peptide sequences searched from the reference genome databases of sorghum, rice, foxtail millet and maize. ZmDEP1a and ZmDEP1b indicate two copies of ZmDEP1 in maize. Scale bar indicates 0.1 amino acid substitutions per site. **b**, *OsGS3* and *ZmGS3*, the orthologs to *GC1*, were reported in rice and maize, respectively. *SiGC1* and *SvGC1*, the homologous genes to *GC1,* are found in *Setaria viridis* and *Setaria italica*, respectively. Two copies of *PmGC1* were searched in *Panicum miliaceum* while three copies of *TaGC1* were detected in *Triticum aestivum*. The red box shows the highly conserved Gγ-like subunit domain. The green box shows the putative transmembrane domain.

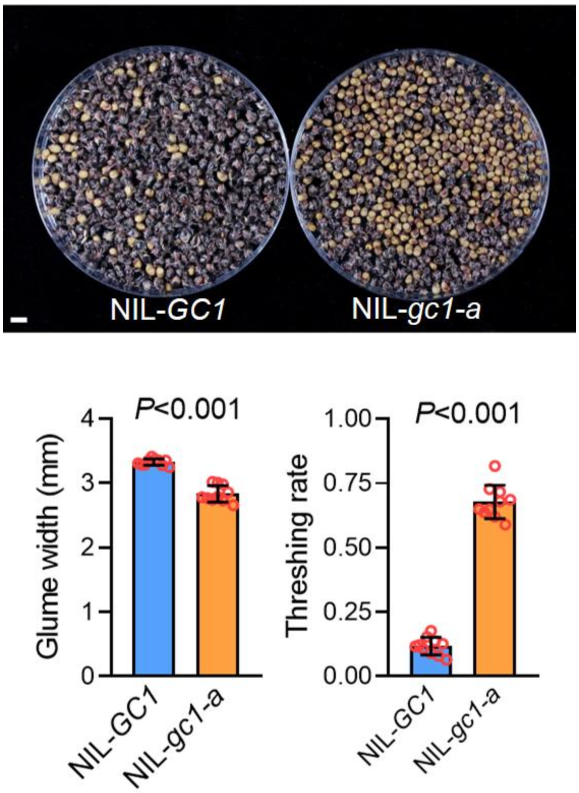

Supplementary Fig. 5. Glume size and threshing rate of NILs.

Threshing rate of NIL-*GC1* and NIL-*gc1-a* after 1000 rpm per minute revolution by a mechanical thresher. Bar = 1 cm. Statistics of glume width and threshing rate of NIL-*GC1* and NIL-*gc1-a*. Phenotypic data are mean ± s.e.m. n = 10 biological replicates. *P*-values were determined by two-tailed unpaired *t*-test. Source data are provided as a Source Data file.

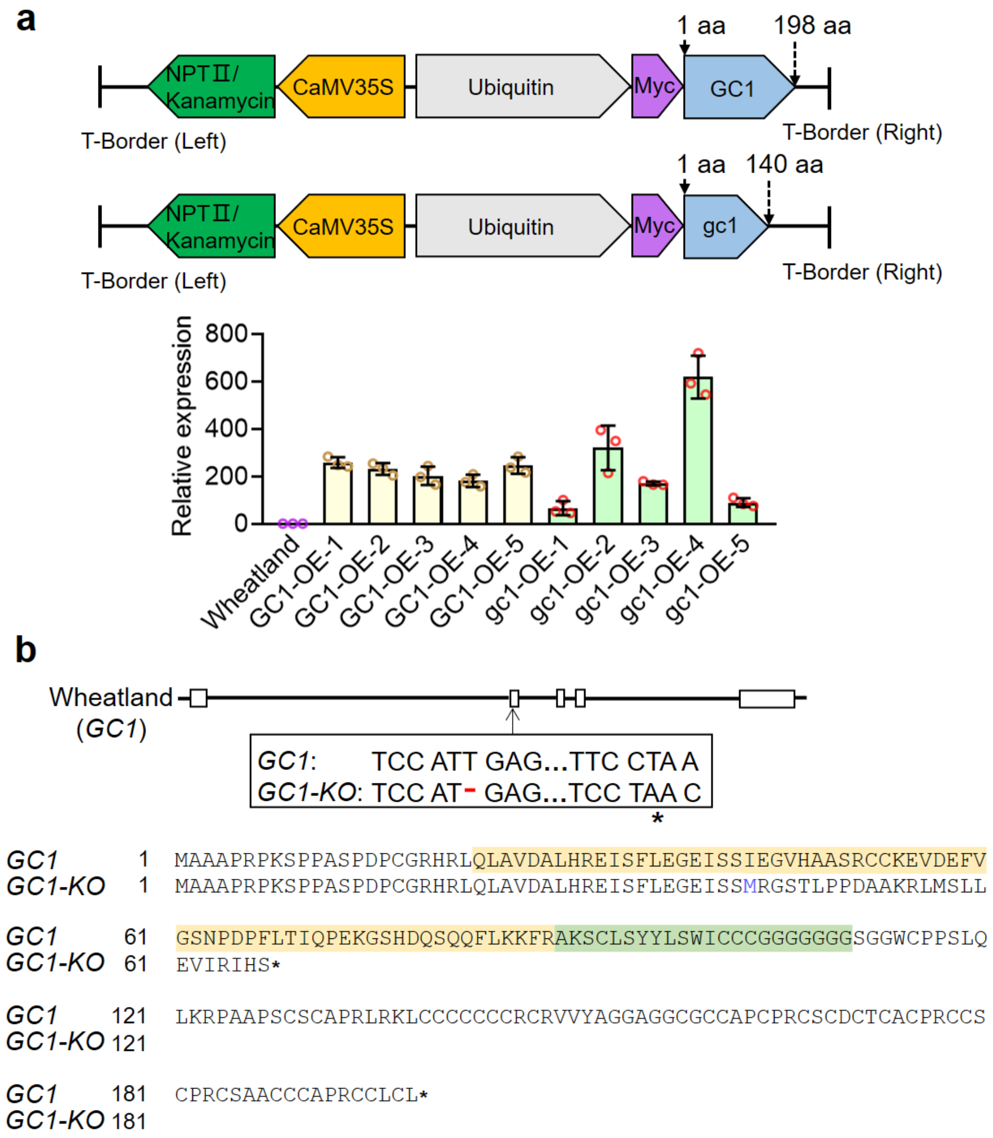

Supplementary Fig. 6. Analysis of overexpression and knockout transgenic plants in sorghum.

a, *GC1*-overexpression (*GC1-OE*) cassette contains the CDS of *GC1* or *gc1*-overexpression (*gc1-OE*) cassette contains the CDS of *gc1* driven by the ubiquitin promoter, with a Myc tag located at the N-terminus. Both constructs were transformed into the sorghum recipient line (Wheatland). gc1 is a truncated version of GC1 with peptide containing 1-140 amino acids (aa). Gene expression of *GC1* alleles in five independent *GC1-OE* and *gc1-OE* lines of the T_0 generation were detected. **b,** Schematic map of the sgRNA target sites and peptide sequence alignment for *GC1* and *GC1-KO* mutants. The mutation site is highlighted by red and blue colors**.** Yellow color shows Gγ subunit-like domain. Green color shows predicted transmembrane domain. The asterisks show stop codon. Source data are provided as a Source Data file.

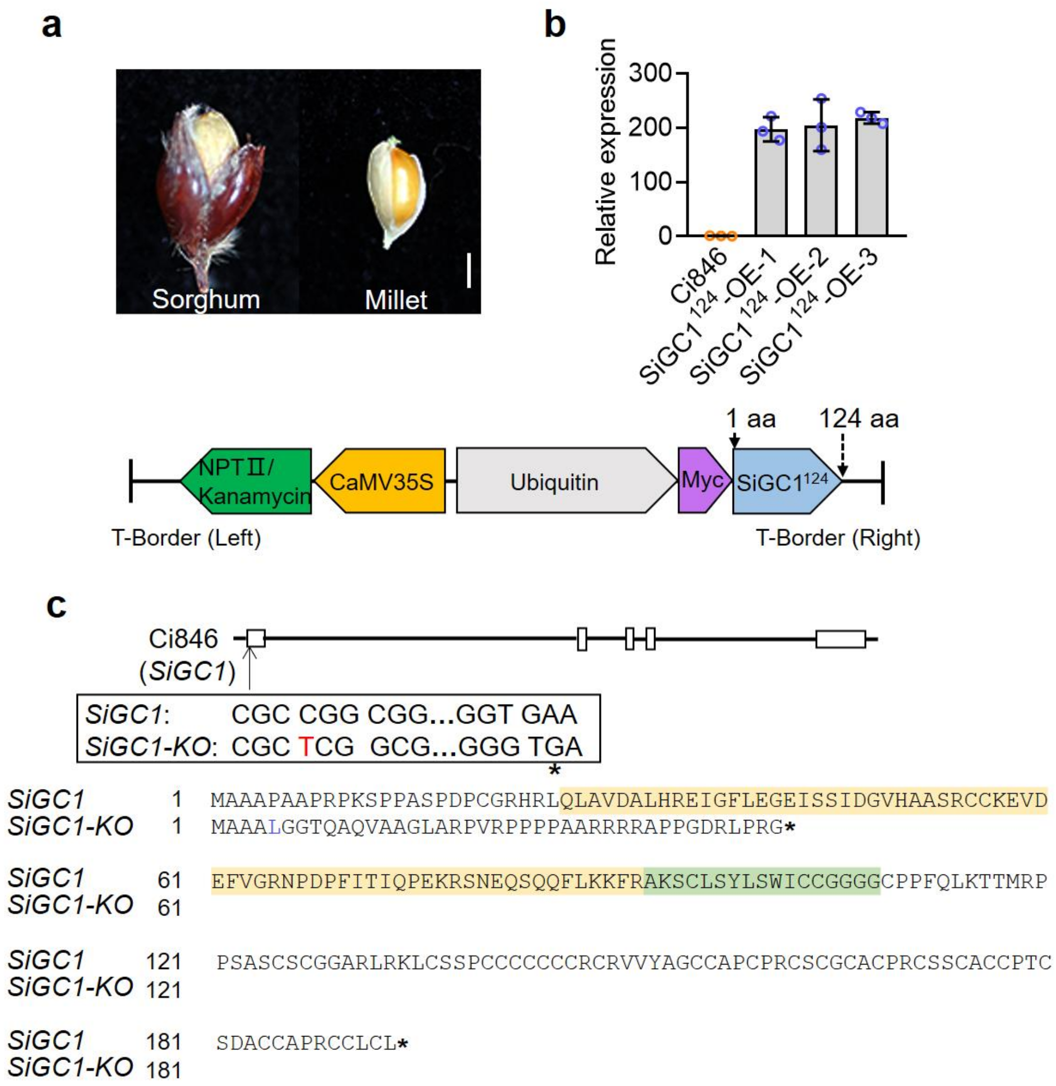

Supplementary Fig. 7. Analysis of overexpression and knockout transgenic plants in millet.

a, Morphology of the mature spikelet of sorghum and millet. Bar = 0.1 cm. **b,** *SiGC1*^{124}-overexpression (*SiGC1*^{124}*-OE*) cassette contains the cDNA of intact Gγ-like subunit of *SiGC1* with a Myc tag at the N-terminus, driven by the ubiquitin promoter, was transformed into the millet recipient line (Ci846). *SiGC1*^{124} expression in three independent *SiGC1*^{124}*-OE* plants of the T_0 generation was detected. Data are mean ± s.e.m. aa, amino acids**. c,** Schematic map of the sgRNA target sites and peptide sequence alignment for *SiGC1* and *SiGC1-KO* mutants. The mutation site is highlighted by red and blue colors**.** Yellow color shows Gγ subunit-like domain. Green color shows predicted transmembrane domain. The asterisks show stop codon. Source data are provided as a Source Data file.

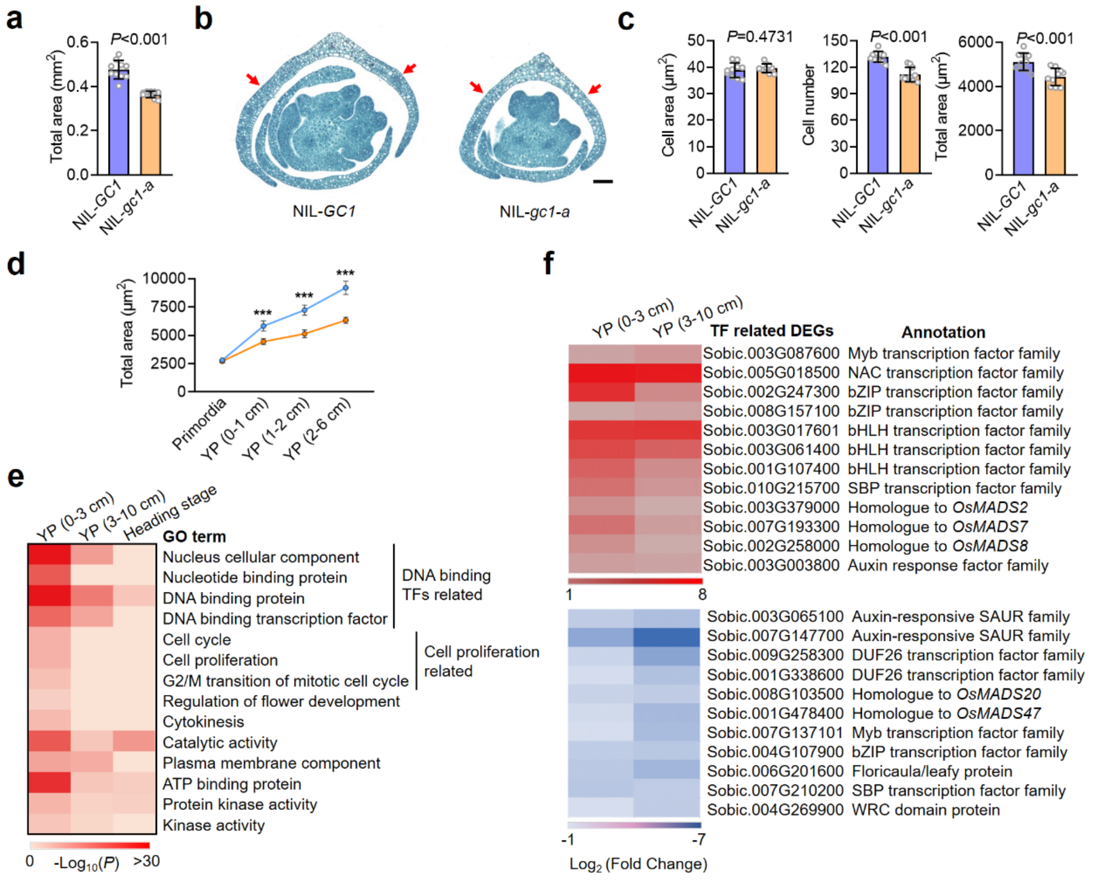

Supplementary Fig. 8. Cell morphology of glumes in the NILs and downstream pathways of *GC1*.

a, Statistics of total cell area in the longitudinal paraffin-section of glumes at the flowering stage. **b,** Glume cell morphology in NIL-*GC1* and NIL-*gc1-a* by cross paraffin-section at flowering stage. Bar = 200 μm. The red arrows show corresponding glume cells. **c,** Statistics of glume cell area, cell number and total area shown in **b**. Data in **a** and **c** are mean ± s.e.m. n = 10 biological replicates. *P*-values were determined by two-tailed unpaired *t*-test. **d,** Statistics of total area at four young panicle developmental stages. YP, Young panicle. Data are mean ± s.e.m. n = 10 biological replicates. *P*-values were determined by multiple two-tailed unpaired *t*-test. *** Significant probability level at $P < 0.001$. **e**, Overrepresented GO categories of downstream DEGs of GC1. TF, Transcription factor. *P*-values were determined by Fisher's exact test. The significantly enriched GO terms were determined by the adjusted *P*-value < 0.05 as the threshold. **f**, Downstream DEGs related to DNA binding TFs pathway were both appeared in 0-3 cm and 3-10 cm young panicles. Red color represent downregulated genes in NIL-gc1-a while blue color show upregulated genes in NIL-*gc1-a*. Fold change was calculated for NIL-*GC1* samples compared with NIL-*gc1-a* samples. Source data are provided as a Source Data file.

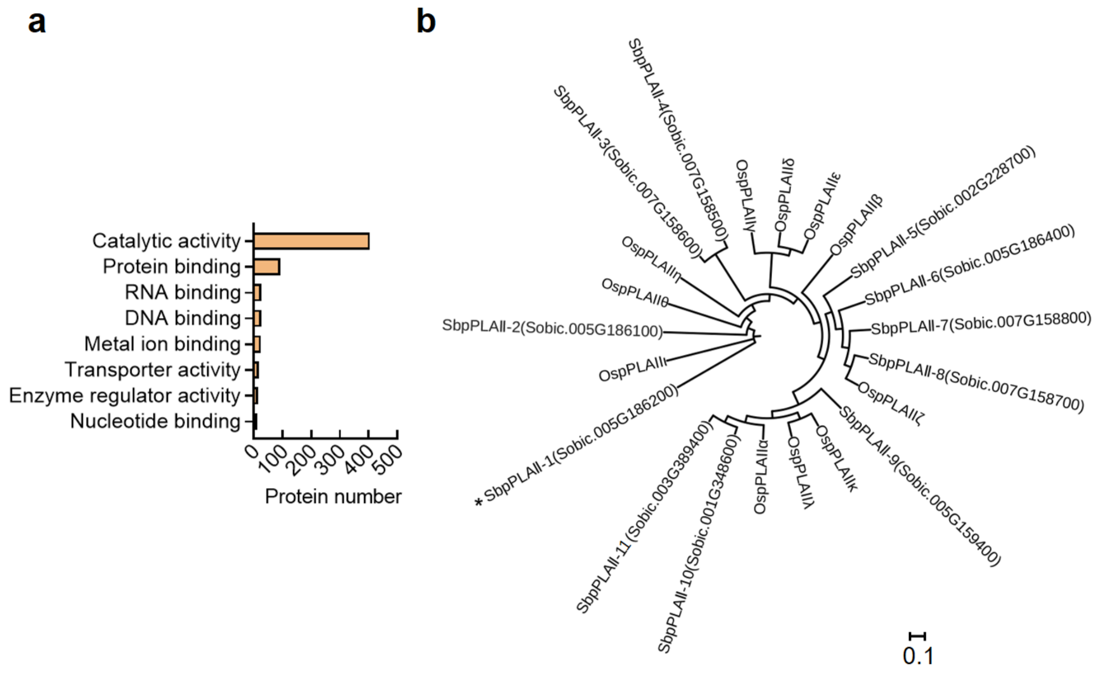

Supplementary Fig. 9. Interacting proteins detected by IP-MS and phylogenetic tree of SbpPLAII subfamily.

a, Gene Ontology-Molecular function (GO-MF) analysis of interacting proteins with Myc tagged gc1 by IP-MS assay. See details in Table S6. **b,** The maximum-likelihood phylogenetic tree based on peptide sequences of 11 sorghum SbpPLAII proteins and 11 rice OspPLAII proteins. The asterisk indicates the SbpPLAII-1. Scale bar indicates 0.1 amino acid substitutions per site.

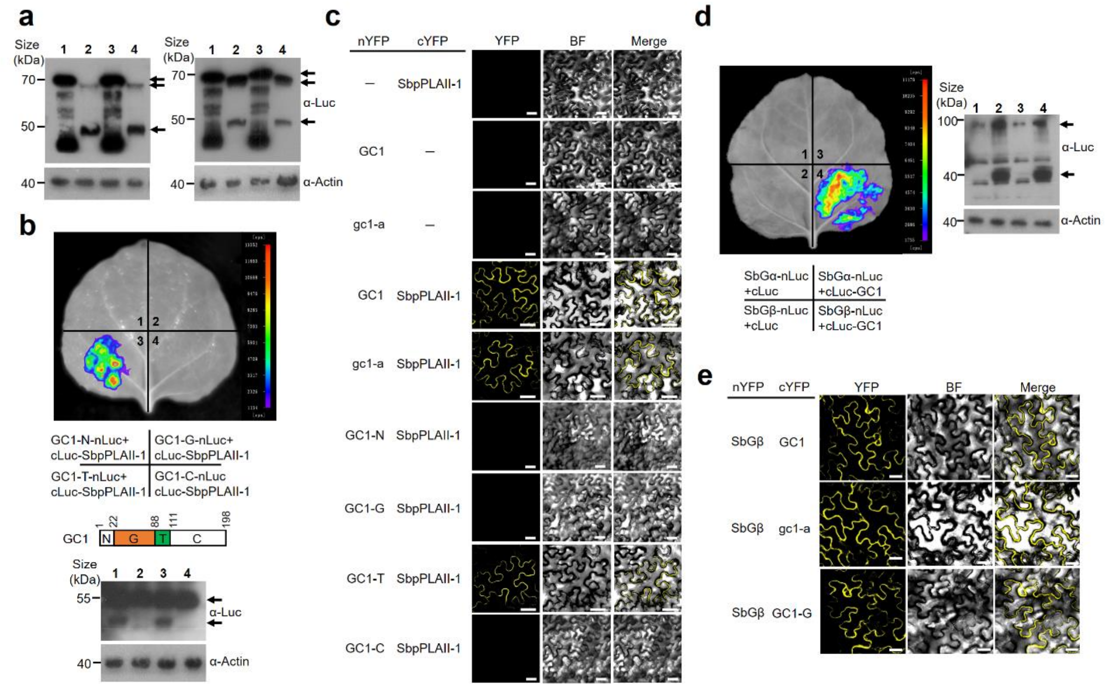

Supplementary Fig. 10. Interactions between GC1, gc1-a and SbpPLAII-1 and SbGβ.

a, The detection of nLuc-tagged GC1 (or gc1-a) and cLuc-tagged SbpPLAII-1 in LCI assays. **b,** Interactions of nLuc-tagged various domain-based versions of GC1 along with cLuc-SbpPLAII-1 by LCI assays. **c,** Interactions between GC1 (or gc1-a) and SbpPLAII-1 in the BiFC assays. nYFP-tagged GC1, gc1-a and domain-based versions of GC1 along with cYFP-SbpPLAII-1 were co-transformed into the tobacco leaves. Bar = 100 μm. **d,** LCI assays show that GC1 protein interacted with SbGβ subunit. The sorghum G protein α subunit (SbGα) and the empty cLuc were used as negative controls. The nLuc-tagged and the cLuc-tagged proteins were detected by anti-luciferase (anti-Luc) antibodies in **a**-**c**. Plant actin was used as the loading control. **e,** BiFC assays show the Gγ-like subunit of GC1 (GC1-G) interacted with sorghum G protein β subunit (SbGβ). Bar = 100 μm. Experiments in **b**-**e** were performed three times.

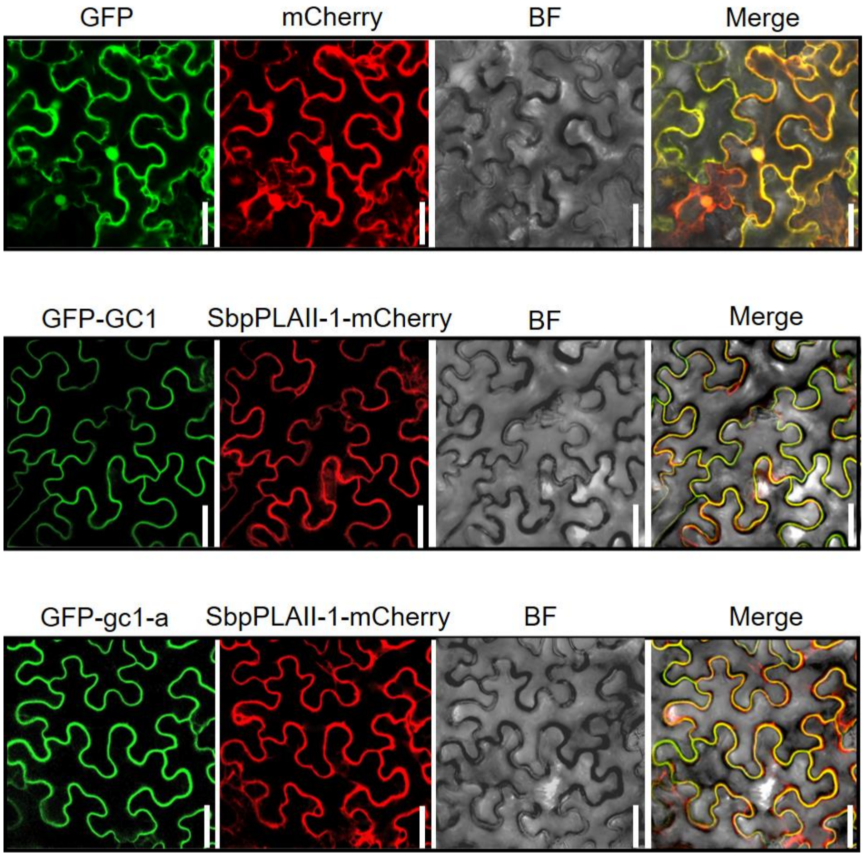

Supplementary Fig. 11. Subcellular localization of GC1, gc1-a and SbpPLAII-1.

GFP-GC1, GFP-gc1-a and mCherry-SbpPLAII-1 proteins are all localized to the cell membrane in *N. benthamiana* leaf cells. Bar = 100 µm. Three repeats were performed.

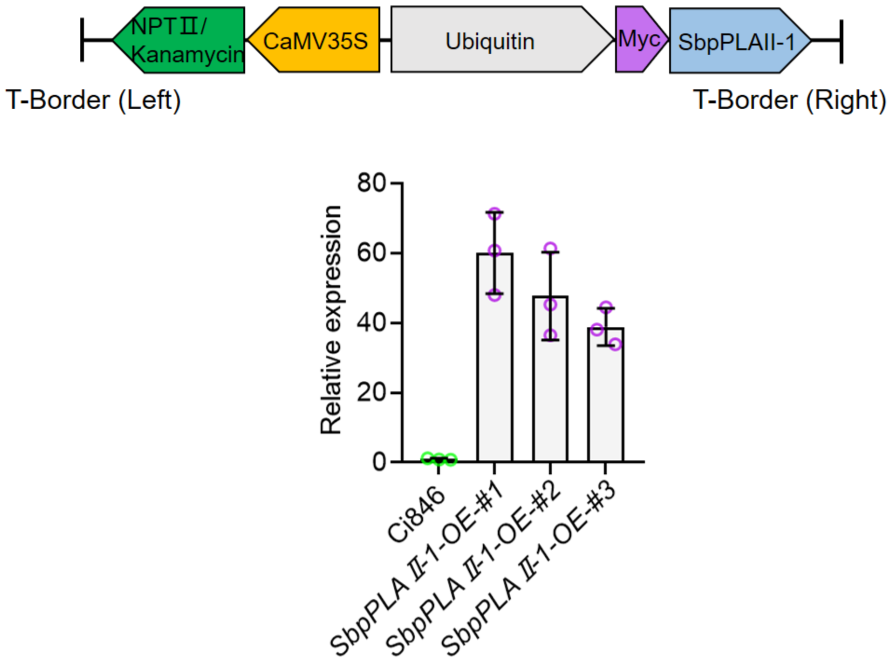

Supplementary Fig. 12. Analysis of *SbpPLAII-1-OE* transgenic plants in millet.

SbpPLAII-1-overexpression (*SbpPLAII-1-OE*) cassette contains the cDNA of *SbpPLAII-1* with a Myc tag driven by the ubiquitin promoter, was expressed into the millet recipient line (Ci846). Gene expression levels of *SbpPLAII-1* in three independent *SbpPLAII-1-OE* millet transgenic plants of the T_0 generation were detected by qPCR. Source data are provided as a Source Data file.

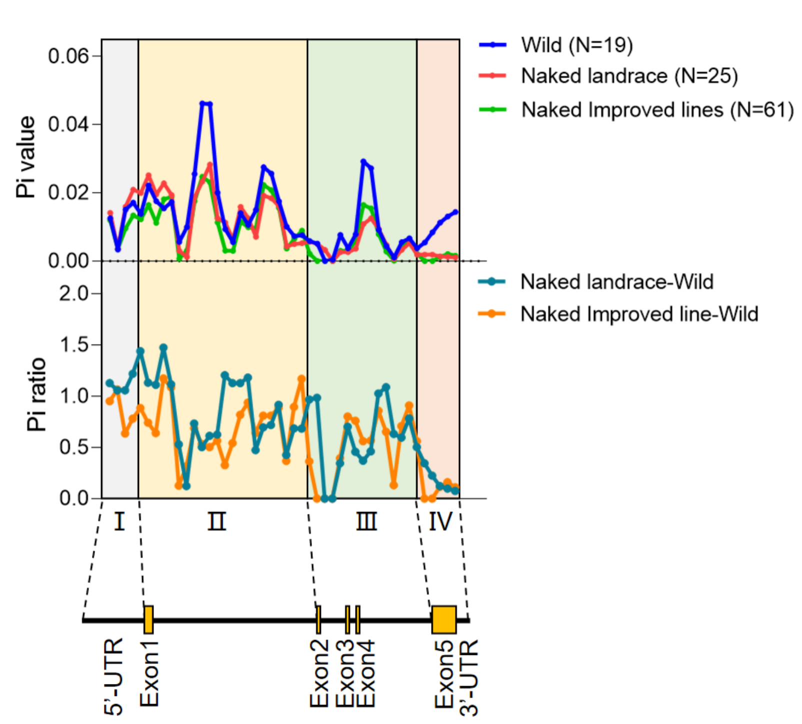

b

Genomic region	I	II	III	IV
Tagima's *D* (Wild)	0.310	0.172	-0.099	-0.311
Tagima's *D* (Naked landrace)	0.593	-0.967	-0.873	-2.539**
Tagima's *D* (Naked improved)	0.196	-1.317	-1.075	-2.751**
Fst (Wild-Naked landrace)	0.070	0.184	0.164	0.288
Fst (Wild-Naked improved)	0.067	0.195	0.165	0.340
Length	886 bp	2446 bp	1637 bp	511 bp

Supplementary Fig. 13. DNA polymorphism and selection analysis of *GC1* gene.

a, A broad profile of nucleotide diversity pi (π) value and pi ratio with variation sites from the genomic segment of *GC1* gene. The selective sweep window size is 200 bp with a step size of 100 bp. Region I, II, III and IV indicate 5'UTR, exon1+intron1, exon2 to intron4 and exon5+3'UTR of *GC1*, respectively. Pi ratio were calculated from the proportions of naked landrace group or naked improved group divided by wild sorghum group. N, number of sorghum accessions. **b**, Statistics of Tajima' *D* tests and fixation index *Fst* values in I, II, III and IV genomic region following the same accessions. ** Significant probability level at $P < 0.01$. Source data are provided as a Source Data file.

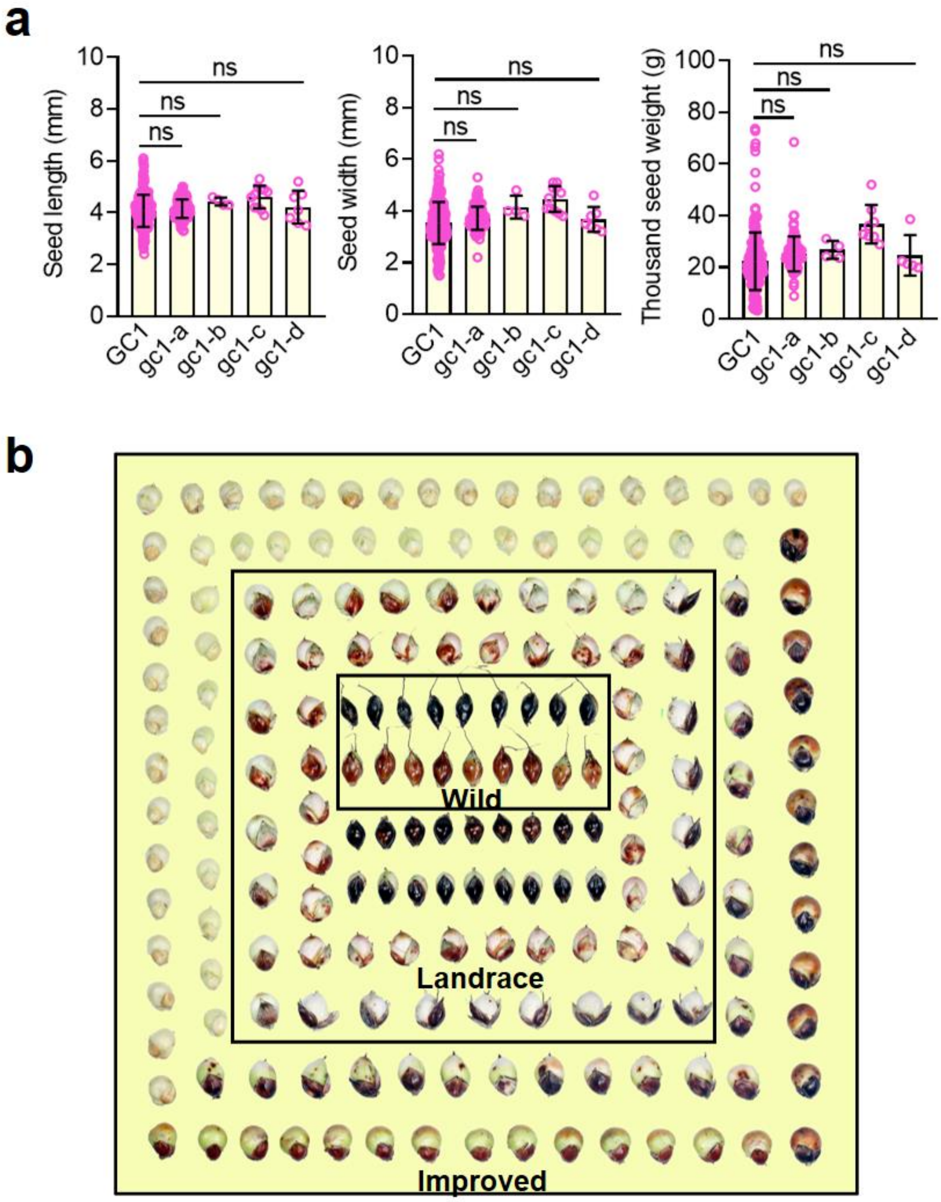

Supplementary Fig. 14. ***GC1*** **haplotypes confer minor effects on grain yield-related traits in diverse sorghum germplasms.**

a, Five *GC1* haplotypes were not significantly associated with grain yield-related traits in the 482 sorghum accessions. Data are mean ± s.e.m. *P* values were determined by one-way ANOVA with Tukey's multiple comparisons test. **b,** The grain morphology of diverse sorghum germplasms. Source data are provided as a Source Data file.
