# Supplementary material for: Natural variation in Glume Coverage 1 causes naked grains in sorghum
Source: Nat Commun. 2022 Feb 25;13:1068. doi: 10.1038/s41467-022-28680-3 (PMC8881591; doi:10.1038/s41467-022-28680-3)
Supplement: Supplementary file 3 — Description of Additional Supplementary Files [file 41467_2022_28680_MOESM3_ESM.pdf]

Description of Additional Supplementary Files

File Name: Supplementary Data 1
Description: Information and phenotype evaluation of 915 diverse sorghum accessions.

File Name: Supplementary Data 2
Description: The SNPs (*P*-value < 10^{-6}) associated with glume coverage in the SAP population.

File Name: Supplementary Data 3
Description: Information of genetic variations across 58 Kb *GC1* region in the 57 sorghum inbred lines.

File Name: Supplementary Data 4
Description: DEGs detected in different developing panicle stages.

File Name: Supplementary Data 5
Description: GO_KEGG analysis in different developing young panicle stages.

File Name: Supplementary Data 6
Description: DEGs related to Cyclin-CDK.

File Name: Supplementary Data 7
Description: Interactive proteins of gc1 by IP-MS.

File Name: Supplementary Data 8
Description: Sorghum accessions were used for selection analysis.

File Name: Supplementary Data 9
Description: All primers used in this study.
