# Supplementary figures and images for: Natural variation in Glume Coverage 1 causes naked grains in sorghum
Source: Nat Commun. 2022 Feb 25;13:1068. doi: 10.1038/s41467-022-28680-3 (PMC8881591; doi:10.1038/s41467-022-28680-3)

**Fig. 3a**

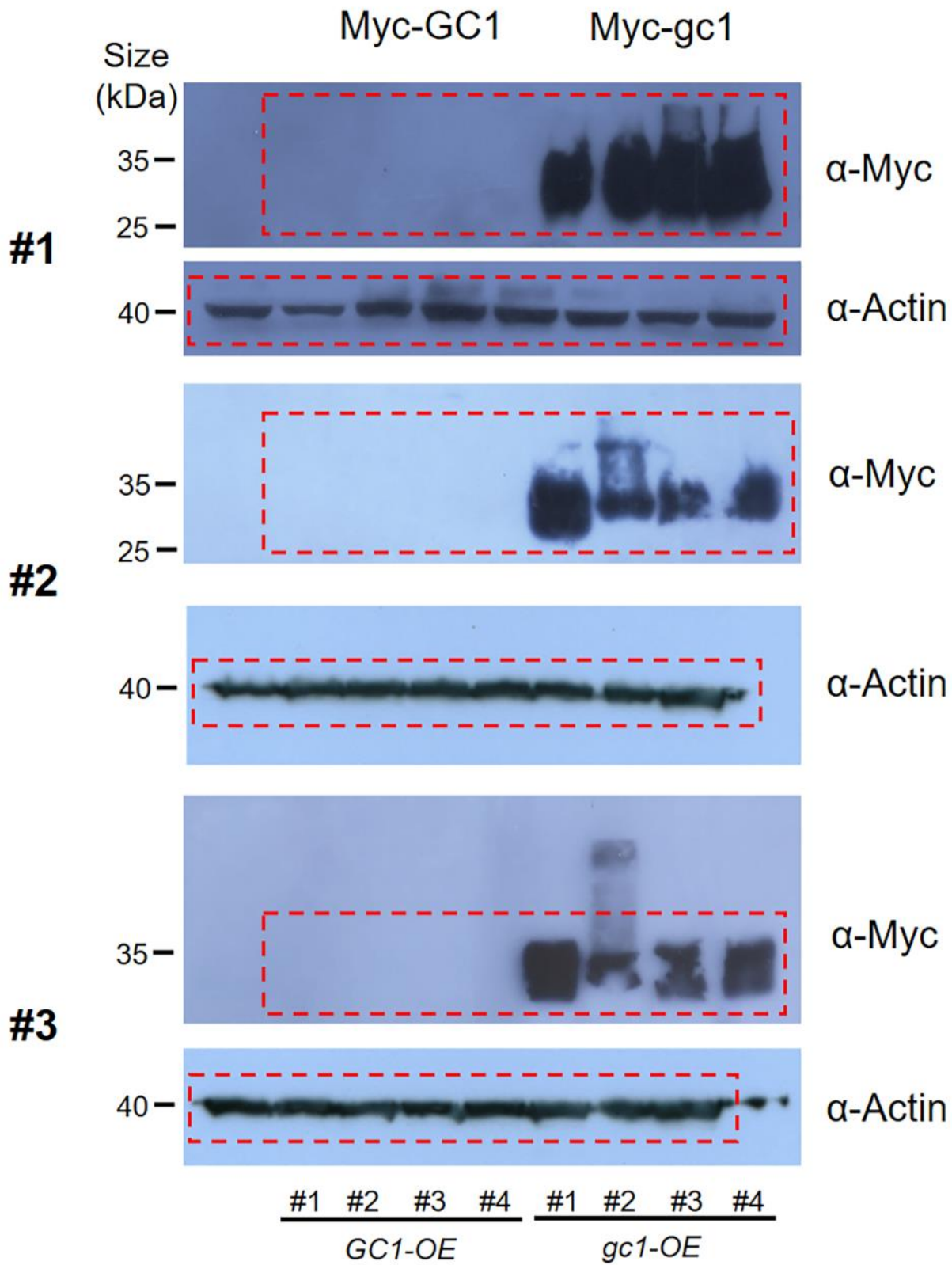

**Fig. 3e**

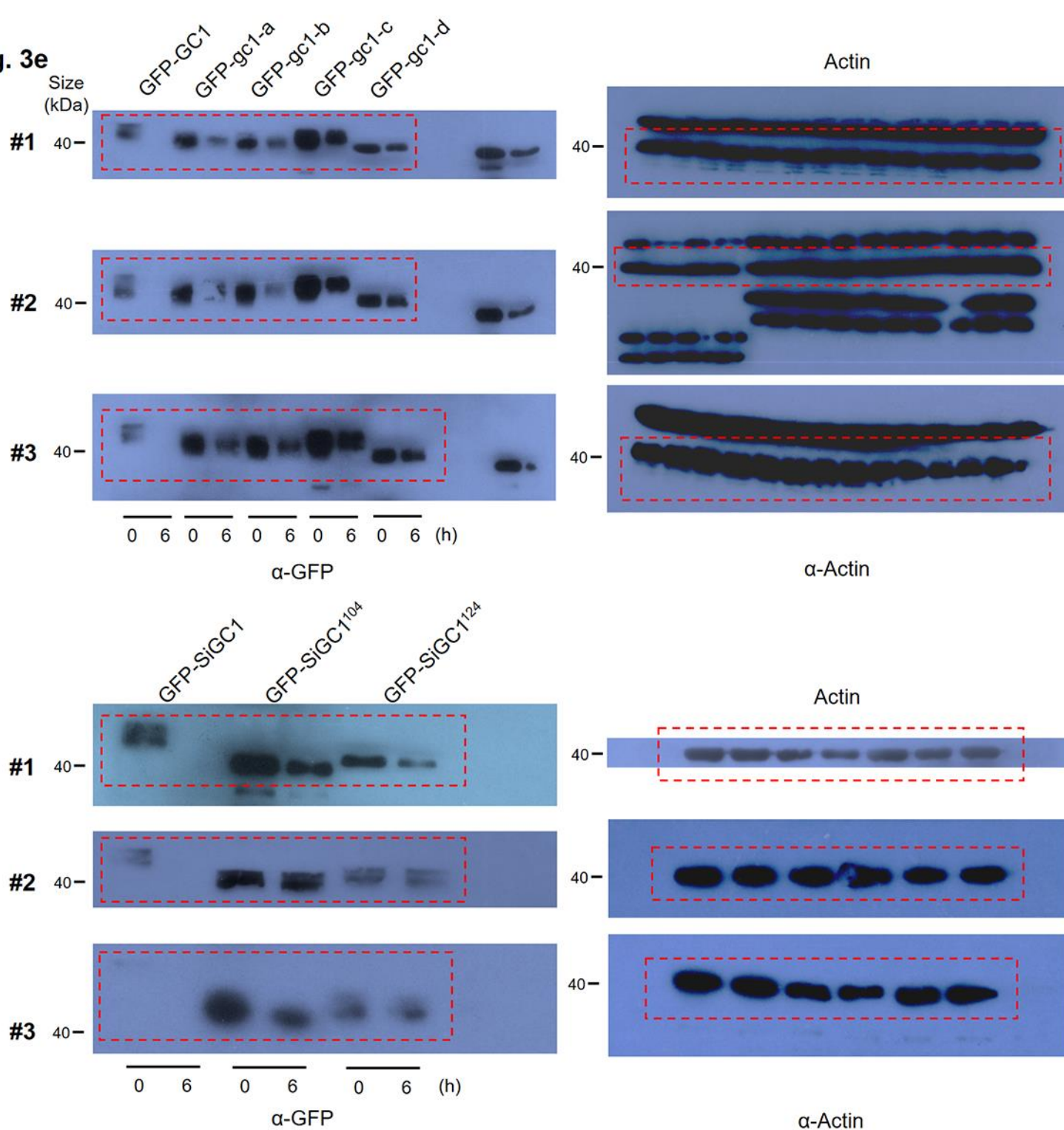

**Fig. 3f**

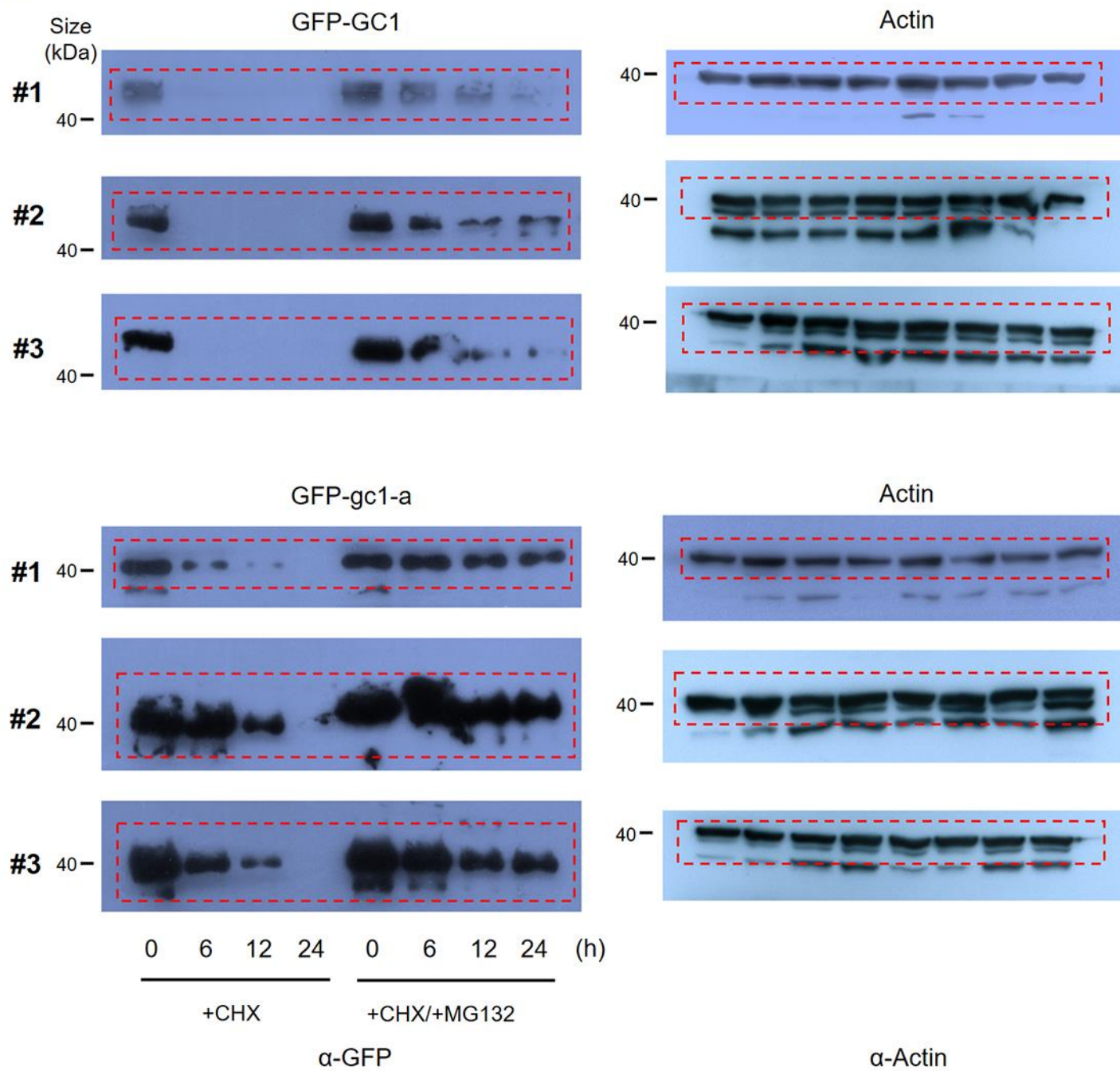

**Fig. 5b**

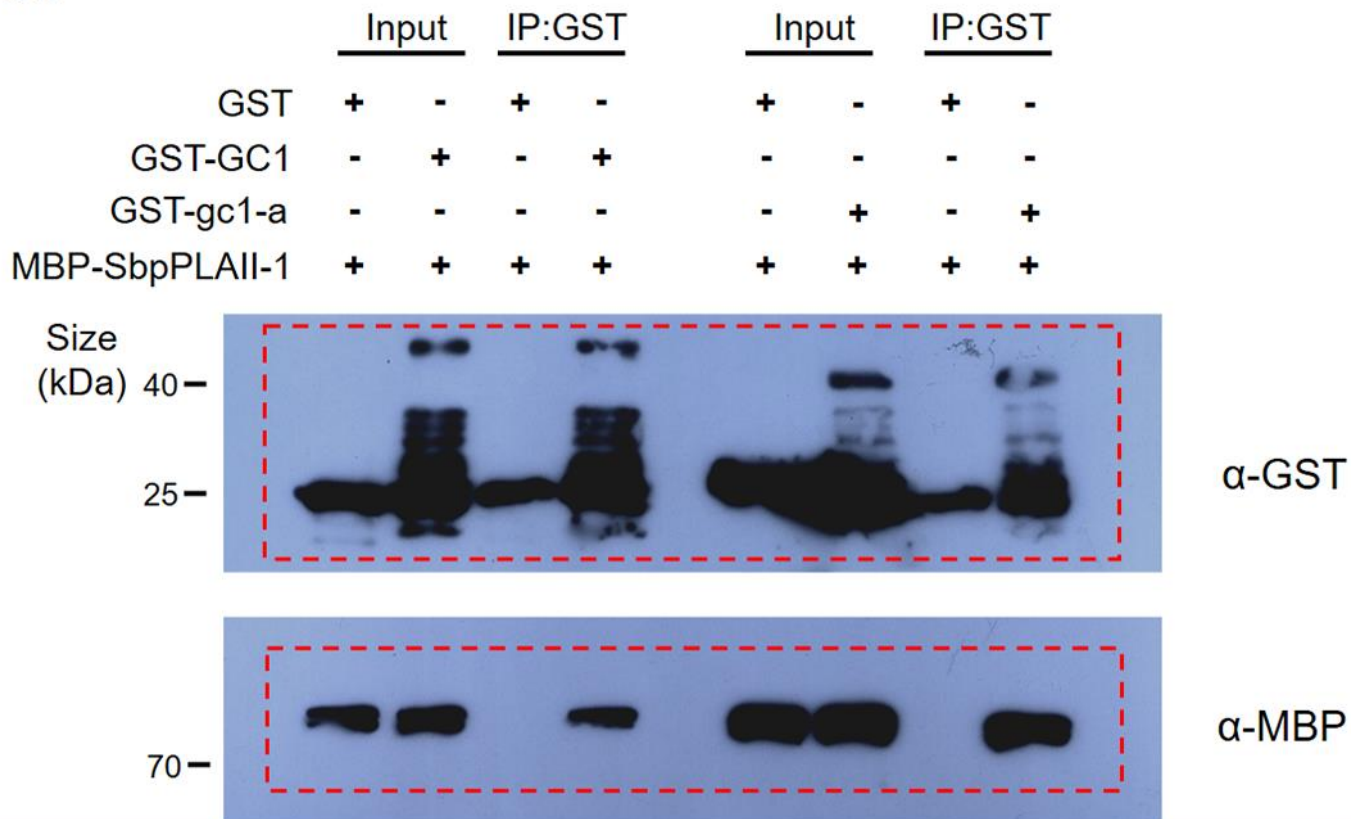

**Fig. 5c**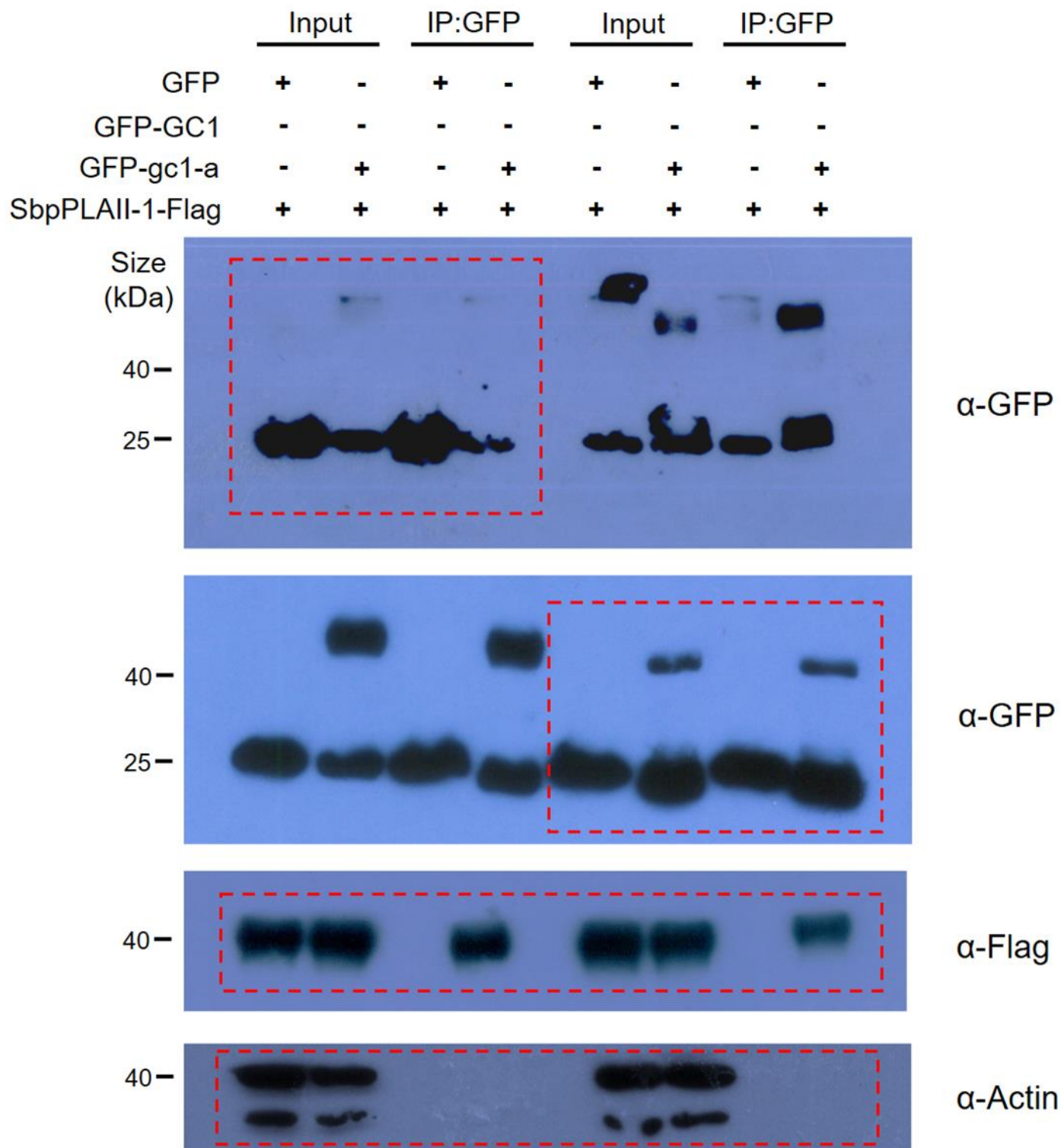

**Fig. 5f**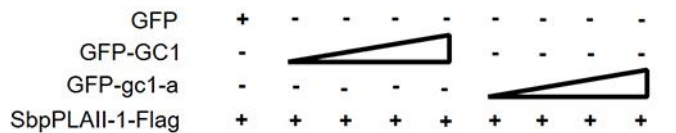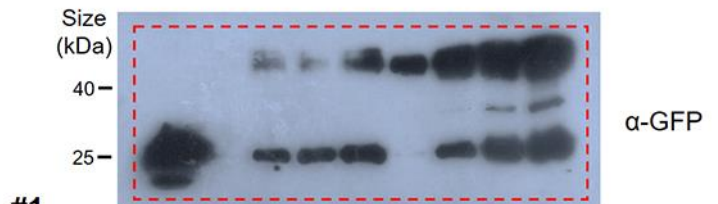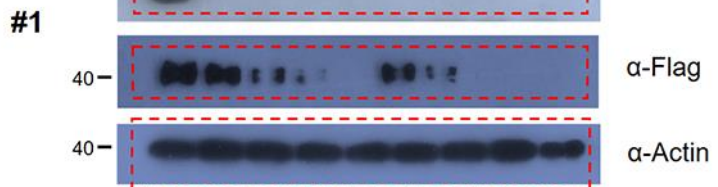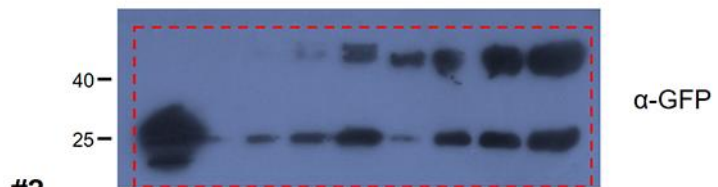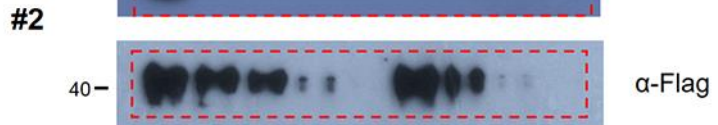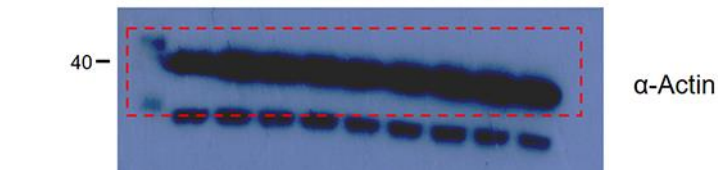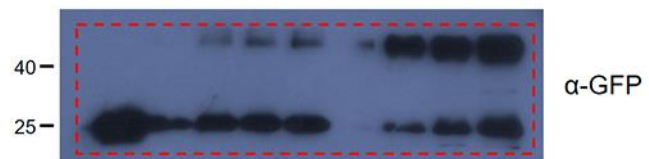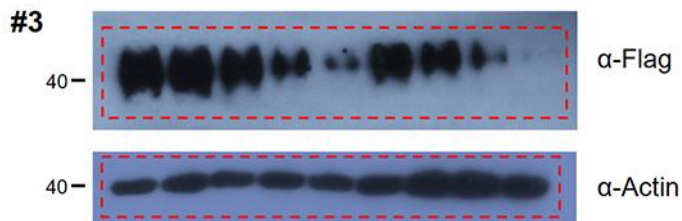

Supplementary Fig. 10a, b and d

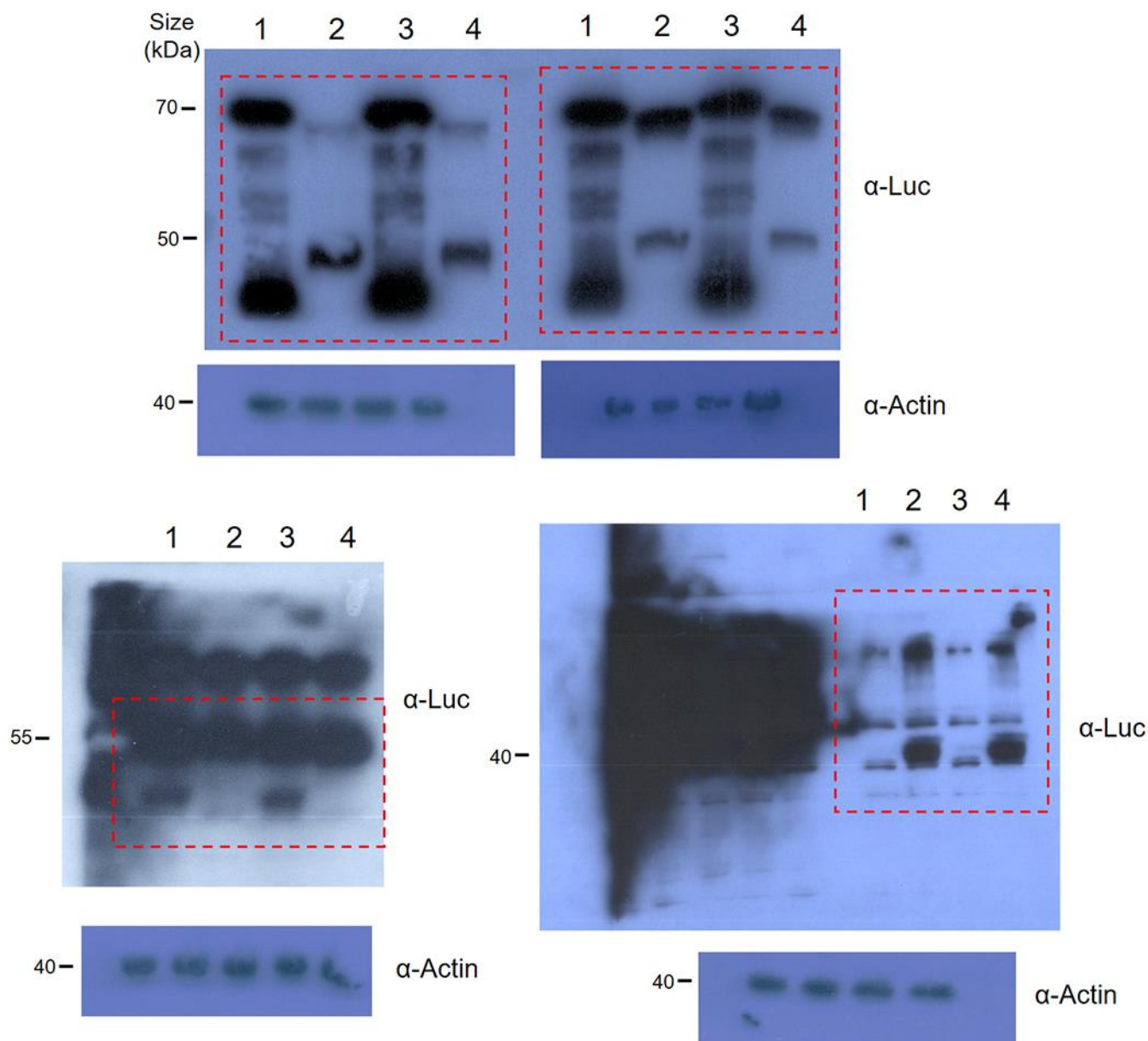

Supplement: Supplementary file 14 — Source data [file 41467_2022_28680_MOESM14_ESM.zip › Uncropped scans of western blots.pdf]
